# Influence of Different Arm Movement Strategies on Subjective Task-Related Perceptions and Walking Outcomes Under Single- and Dual-Task Conditions in Healthy Children Compared to Young Adults

**DOI:** 10.3390/brainsci16040428

**Published:** 2026-04-20

**Authors:** Katharina Borgmann, Matthias Schebeck, Lea Greiwe, Johanna Lambrich, Mathew W. Hill, Thomas Muehlbauer

**Affiliations:** 1 Division of Movement and Training Sciences/Biomechanics of Sport, University of Duisburg-Essen, 45141 Essen, Germany; katharina.borgmann1@uni-due.de (K.B.); m.schebeck@googlemail.com (M.S.); lea.greiwe@stud.uni-due.de (L.G.); johanna.lambrich@uni-due.de (J.L.); 2 Institute of Biomechanics and Orthopaedics, German Sport University Cologne, 50933 Cologne, Germany; 3 School of Psychology and Vision Sciences, University of Leicester, Leicester LE1 7RH, UK; mwh20@leicester.ac.uk

**Keywords:** postural control, locomotion, perception, attentional demand, upper-body strategy, age

## Abstract

**Highlights:**

**What are the main findings?**

- Walking under dual-task conditions negatively affected both subjective task-related perceptions and walking outcomes in children and young adults, but allowing free arm movements mitigated these deteriorations, indicating a compensatory effect.
- Children maintained relatively stable levels of conscious balance processing, whereas young adults showed higher levels and marked changes between walking under single- and dual-task conditions with free arm movements.

**What are the implications of the main findings?**

- Free arm movements appear to represent an effective complementary ‘upper-body strategy’ to support postural control under cognitively demanding walking conditions.
- Young adults possess sufficient attentional and cognitive resources to shift from automated to more conscious postural control when required, while children may rely more strongly on automated processes.

**Abstract:**

**Background/Objectives**: Emerging evidence shows that dual tasking as well as the restriction of arm movements independently lead to detrimental effects on walking performance. However, it is unclear whether the deteriorations are more pronounced when applied together and if children (i.e., due to ongoing maturation processes) perform differently compared to young adults. This study investigated the influence of different arm movement strategies on subjective and objective markers related to beam walking under single-task (ST) and dual-task (DT) conditions in children and young adults. **Methods**: Twenty-six children (age: 11.3 ± 0.6 years) and 30 young adults (age: 23.2 ± 2.8 years) walked three meters on a balance beam with free and restricted (i.e., arms crossed over the chest) arm movements in a random order while concurrently performing a cognitive task (i.e., serial subtractions) or not. Walking outcomes (i.e., gait speed, cadence) were measured and used as objective markers. Self-reported task-related perceptions (i.e., balance confidence, fear of falling, perceived instability, conscious balance processing) were assessed and used as subjective indicators. **Results**: Walking under DT conditions (i.e., main effects of task) detrimentally influenced subjective task-related perceptions and walking outcomes, but using free arm movements (i.e., task × arm interactions) mitigated these deteriorations. Further, children exhibited largely stable levels of conscious balance processing, whereas young adults demonstrated overall higher levels along with pronounced differences between ST and DT walking when arm movements were unrestricted (i.e., group × task × arm interaction). **Conclusions**: These findings indicate that free arm movements seem to constitute a simple yet effective complementary ‘upper-body strategy’ that enhances postural control during a cognitively demanding walking task. Further, age differences imply that young adults compensate demanding walking conditions (i.e., DT walking with restricted arms) by elevated conscious processing of balance (i.e., a shift from automated to more conscious attention towards postural control).

## 1. Introduction

In daily life, situations that require the simultaneous processing of dynamic balance and cognitive tasks—commonly referred to dual-task (DT) conditions—are the norm rather than the exception. Typical examples include recalling the agenda for an upcoming meeting while walking to a conference room or talking on a cell phone while crossing a busy street. Research involving healthy young adults has consistently shown that DT conditions combining dynamic balance tasks (e.g., walking) with cognitive tasks (e.g., serial subtraction) lead to decrements in balance performance. For example, Beurskens and Bock [1] as well as Maiocchi et al. [2] reported significant DT-related performance deteriorations compared to single-task (ST) conditions, including reduced gait speed in young adults.

Performance deficits observed under DT conditions are commonly attributed to limited cognitive resources (i.e., “central overload”) [3] and/or to interference arising when two tasks rely on overlapping processing resources [4]. Two prominent theoretical frameworks frequently used to explain DT-related performance deficits are the central processing bottleneck theory (also known as the “single-channel model”) [3,5] and the capacity-sharing model [6]. According to the single-channel model, cognitive operations are processed sequentially, and a bottleneck occurs when multiple tasks simultaneously require substantial cognitive resources. Otherwise, the capacity-sharing model proposes a common pool of processing resources that can be flexibly allocated across tasks. When a larger proportion of resources is devoted to one task, fewer resources remain available for the other, resulting in performance decrements in one or both tasks.

While the capacity-sharing model emphasises the allocation of cognitive resources across tasks, motor strategies may also influence how these resources are utilised during DT performance. Supporting this notion, recent studies [7,8] have demonstrated that performance deterioration was less pronounced when arm movements are permitted under DT conditions. In fact, da Silva Costa et al. [8] investigated adults and reported greater step speed during DT walking on a balance beam when arm movements were unrestricted compared to when they were constrained. Free arm movements may serve as a compensatory mechanism by distributing body mass, increasing the moment of inertia, generating restoring torques, and shifting the centre of mass over the base of support [9,10,11], thereby enhancing postural control.

Although the work of da Silva Costa and colleagues [8] provides valuable insight, it was conducted exclusively in adults. Consequently, potential differences related to immature postural control mechanisms in children [12,13,14,15,16] and the ongoing development of cognitive processing capacities [17,18] were not considered. Concerning the development of postural control, Hirabayashi and Iwasaki [14] showed that proprioceptive function developed early, followed by visual function, which reaches adult level around the age of 15 years. Moreover, vestibular function develops later and remains less mature than in adults even at this age. Consistent with these findings, several studies have shown that children exhibit greater postural sway than young adults, particularly when standing with eyes closed or on a moving surface [12,13,15,16].

With respect to cognitive development, Perlman and Pelphrey [18] compared emotional brain connectivity between children and adults and found an increase in effective connectivity (i.e., a neural mechanism for the development of affective self-regulation) with age. In this context, we sought to extend the current level of knowledge concerning the compensatory effect of free arm movements on DT-related performance deficits—exclusively obtained in adults—by directly comparing children and young adults. Based on previous studies in adults [7,8], we hypothesised that (1) walking under DT conditions would detrimentally influence both subjective task-related perceptions and walking outcomes, (2) these detrimental effects would be lower when free arm movements are allowed, but (3) the compensatory effect of free arm movements may differ between children and young adults, with smaller effects expected in children, potentially due to differences in postural and cognitive development.

## 2. Materials and Methods

### 2.1. Sample Size Estimation and Participants

Research sample size estimation for analysis of variance (ANOVA) with repeated measures was performed based on findings reported in previous studies [7,8] that investigated the effect of free versus restricted arm movement strategies during ST and DT walking. G*Power 3.1.9.7 [19] was used (input parameters: *f* = 0.25, *α* = 0.05, 1 − *β* = 0.80, no. of groups = 2, no. of measurements = 4, correlation among repeated measures: *r* = 0.20, non-sphericity correction = 1) and revealed that a total sample size of 38 participants would be necessary to identify statistically significant within–between interactions. Twenty-six children (14 females, 12 males) aged 11.3 ± 0.6 years were recruited from a secondary school, and 30 young adults (15 females, 15 males) aged 23.2 ± 2.8 years were enrolled from the student population of the host institution. Inclusion criteria were age <13 and 20–30 years. We excluded individuals who reported musculoskeletal dysfunction, neurological impairment, orthopaedic disorder or a recent injury. All participants voluntarily enrolled in this study and provided written informed consent (as well as the children’s parents), and the Human Ethics Committee at the University of Duisburg-Essen, Faculty of Educational Sciences approved the study protocol (approval number: EA-PSY9/24/25032024).

### 2.2. Experimental Procedures

**Figure 1:**
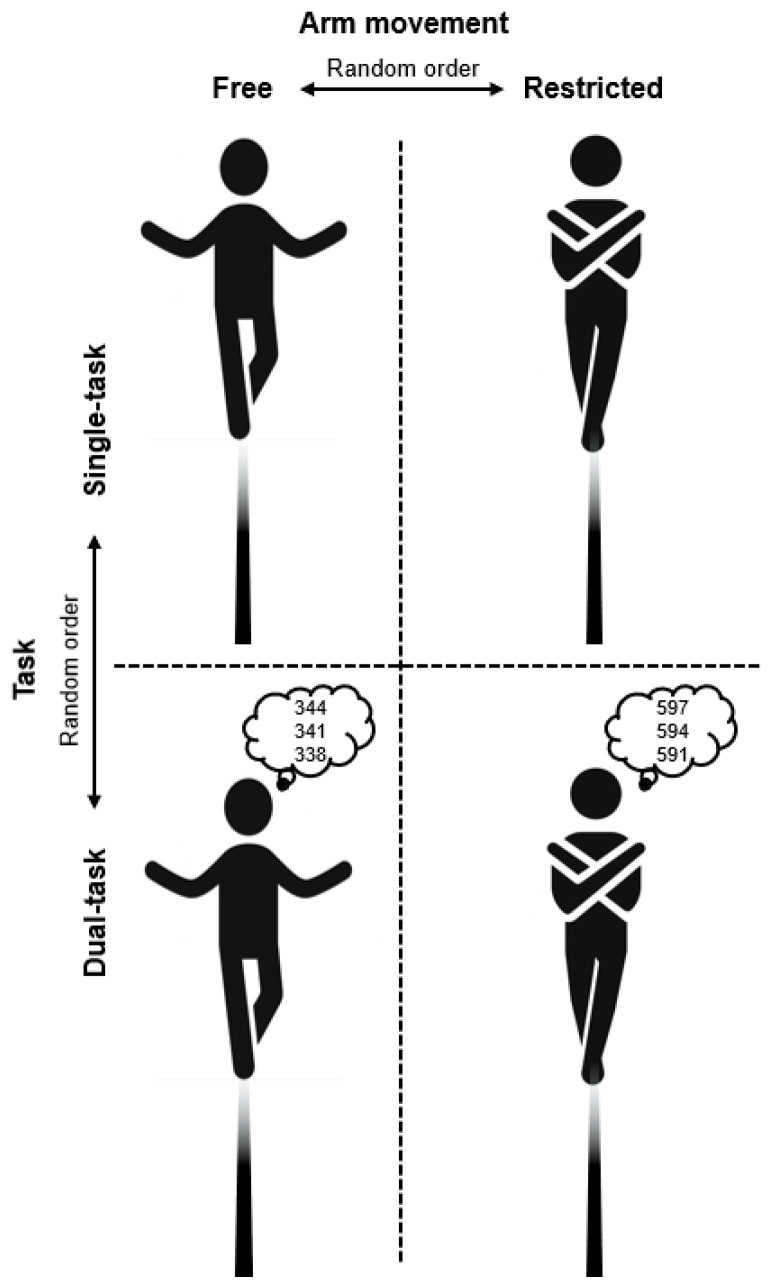
Schematic diagram of the experimental protocol showing the task (single task vs. dual task) and arm movement (free vs. restricted) conditions.

The whole experimental procedure was conducted by the same skilled assessors (graduated sport scientists). For the dynamic balance task, all participants were asked to walk forward across a wooden balance beam (length: 300 cm, width: 4.5 cm, height: 5.0 cm) at a self-selected speed while wearing their own sports shoes under two different arm movement conditions: (i) free arm movements (i.e., arms free to move), and (ii) restricted arm movements (i.e., arms crossed over the chest). Each walk was initiated and terminated on a wooden platform (30 cm × 30 cm) positioned at the beginning and the end of the beam to enable a safe initial and final state. All participants completed one data collection trial per arm movement strategy under ST (motor task only) and DT (motor + cognitive task) conditions. The order of the four conditions was randomised, both in terms of the arm and task conditions (Figure 1). Prior to data collection, participants completed a practice trial for each condition to familiarise themselves with the four conditions. When performing the ST conditions, participants were instructed as follows: *“Walk forward across the beam without losing your balance”*, while under DT conditions, the instruction was: *“Walk forward across the beam without losing your balance and perform as many serial three subtractions as possible”*. When the DT methodology was applied, participants were instructed to give equal priority to both tasks in order to create real-life conditions [20].

The cognitive task consisted of a serial subtraction task in which participants verbally subtracted a value of three consecutively from a randomly selected starting number between 300 and 900 provided by the experimenter [21]. Incorrect responses were recorded as errors. When participants resumed the task correctly following an error, only the initial miscalculation was counted. The total number of subtractions minus the number of subtraction errors was registered and divided by walking time, with higher scores per second (n/s) indicating better cognitive task performance.

### 2.3. Assessment of Subjective Task-Related Perceptions

Prior to each trial, participants rated their balance confidence using a visual analogue scale (VAS) that ranged from 0 (“Not at all confident”) to 10 (“Completely confident”) [22,23], with higher values indicating greater balance confidence. Following each trial, participants reported their fear of falling experienced during the trial using a VAS from 0 (“Not at all fearful”) to 10 (“Completely fearful”) [24,25], with higher scores indicating greater fear of falling. Participants also rated their perceived instability during the trial on a VAS, with scores ranging from 0 (“Completely stable”) to 10 (“So instable that I would fall”) [26,27], with higher values indicating greater perceived instability. In addition, conscious processing of balance was evaluated using a four-item questionnaire. The items were: (1) “I always try to think about my balance when I perform this task”; (2) “I am aware of how my mind and body are functioning when performing this task”; (3) “I am aware of how I look when performing this task”; and (4) “I am concerned about my movement style when performing this task.” Responses were scored on a six-point Likert scale ranging from 1 (“strongly disagree”) to 6 (“strongly agree”) [28]. A total score ranging between 4 and 24 points was calculated by summing responses across items, with higher scores indicating greater conscious processing of balance.

### 2.4. Assessment of Walking Outcomes

The time (s) taken to cross the 300 cm long wooden beam was recorded (resolution: 0.01 s) using a measuring system that consisted of two double-light barriers (WITTY TIMER, Microgate Srl, Bolzano, Italy). The measurement started and ended automatically when the barrier at the beginning and the end of the beam was passed, respectively. Step number (*n*) was counted visually by the same experimenter while the participants crossed the beam starting and terminating the walk on a wooden platform (30 × 30 cm) that was placed at the beginning and the end of the beam. A trial was discarded and repeated a maximum of three times if participants lost their balance at any point during the trial (i.e., touched the ground) or removed their arms from the crossed position during the restricted arm movement condition. This situation occurred in two to three cases per age group. For further analyses, gait speed (m/s) was calculated by dividing the walking distance (m) by the time (s) needed. Moreover, cadence (*n*/s) was computed from the quotient of the step number (*n*) and the time (s) required to cross the beam.

### 2.5. Statistical Analyses

Data were analysed using JASP version 0.19.3.0 (Amsterdam, The Netherlands). For all analyses, assumptions of normality (Shapiro–Wilk’s test) and homogeneity of variance (Levene’s test) were checked and met prior to conducting parametric analyses. A series of separate mixed-model ANOVAs were performed to examine the between-subject effect of age group (×2 [children vs. young adults]) and the within-subject effects of task conditions (×2 [single task vs. dual task]) and arm movement strategies (×2 [free vs. restricted]). Where significant interactions or main effects were detected, *post-hoc* analyses using Bonferroni-adjusted α determined the location of any differences. For the ANOVAs, effect sizes were expressed as partial eta-squared ($\eta_{p}^{2}$) and categorised as small (0.02 ≤ $\eta_{p}^{2}$ ≤ 0.12), medium (0.13 ≤ $\eta_{p}^{2}$ ≤ 0.25), or large ($\eta_{p}^{2}$ ≥ 0.26). For the *post hoc* analyses, effect sizes were reported as Cohen’s *d* using the appropriate formulation for repeated-measures comparisons and interpreted as trivial (0 ≤ *d* ≤ 0.19), small (0.20 ≤ *d* ≤ 0.49), moderate (0.50 ≤ *d* ≤ 0.79), or large (*d* ≥ 0.80). Additionally, 95% confidence intervals were reported for all relevant pairwise comparisons. The alpha level for all tests was set *a priori* at *p* ≤ 0.05.

## 3. Results

**Figure 2:**
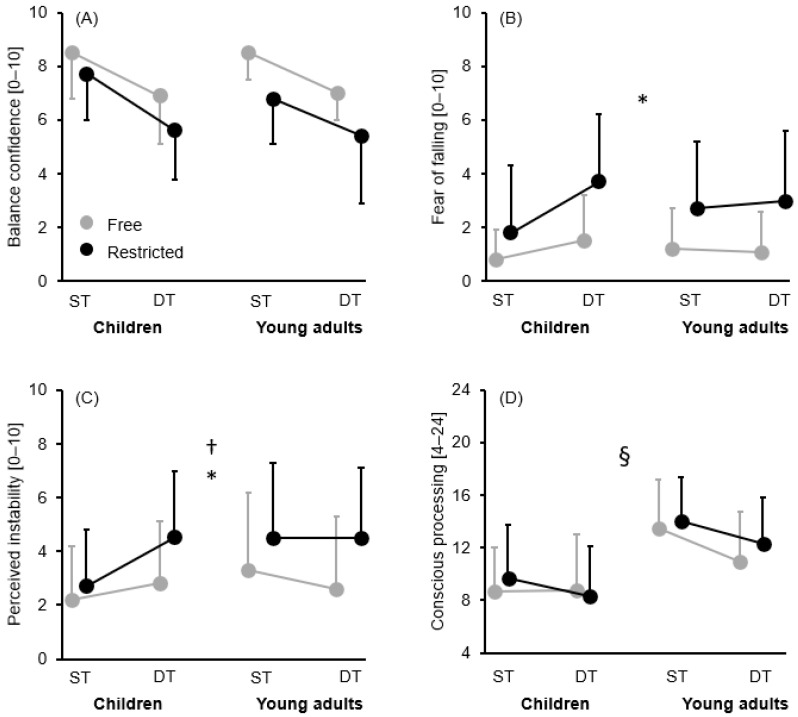
Group mean ± standard deviation values for the subjective task-related perception outcomes: (**A**) balance confidence; (**B**) fear of falling; (**C**) perceived instability; (**D**) conscious balance processing by task (single task vs. dual task) and arm movement (free vs. restricted) conditions. † Represents a significant task × arm interaction (*p* ≤ 0.05). * Represents a significant difference group × task interaction (*p* ≤ 0.05). § Represents a significant group × task × arm interaction (*p* ≤ 0.05). ST = single task, DT = dual task.

**Figure 3:**
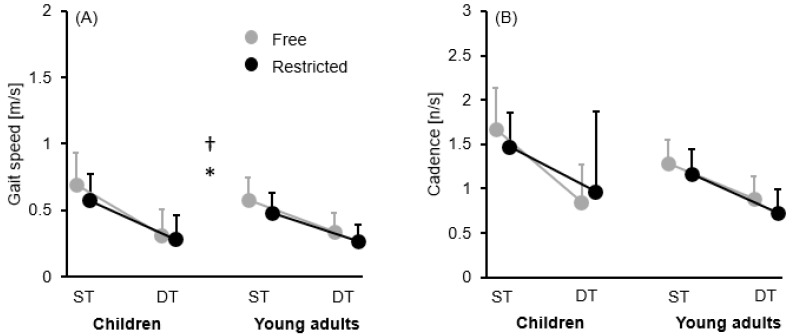
Group mean ± standard deviation values for the walking outcomes: (**A**) gait speed; (**B**) cadence by task (single task vs. dual task) and arm movement (free vs. restricted) conditions. † Represents a significant task × arm interaction (*p* ≤ 0.05). * Represents a significant difference group × task interaction (*p* ≤ 0.05). ST = single task, DT = dual task.

Table 1 and Figure 2A–D and Figure 3A,B present the descriptive statistics consisting of group mean ± standard deviation values, while Table 2 provides the ANOVA outputs for all assessed variables.

### 3.1. Subjective Task-Related Perception

#### 3.1.1. Balance Confidence

There were significant main effects for task and arm, with participants reporting lower balance confidence during the DT (irrespective of arm movement strategy) and the restricted arm movement (irrespective of task) conditions.

#### 3.1.2. Fear of Falling

Again, we detected significant main effects for task and arm, with participants showing greater fear of falling during the DT (irrespective of arm movement strategy) and the restricted arm movement (irrespective of task) conditions. Further, we observed a significant group × task interaction (Figure 2B). Post hoc tests revealed a significant increase in fear of falling during walking under DT compared to ST conditions (irrespective of arm movement strategy) in children (*t* = −3.858, *p* = 0.002, *d* = 0.62, 95% *CI* [−2.203, −0.374]) but not in young adults (*t* = −0.268, *p* = 1.000, *d* = 0.04, 95% *CI* [−0.935, 0.768]).

#### 3.1.3. Perceived Instability

There was a significant main effect of arm, with participants stating higher perceived instability during the restricted arm movement conditions (irrespective of task). Further, we detected a significant task × arm interaction and group × task interaction (Figure 2C). In the first case, *post hoc* tests yielded a significant increase in perceived instability during walking with restricted compared to free arm movements under DT (*t* = −5.210, *p* < 0.001, *d* = 0.71, 95% *CI* [−2.744, −0.853]) but not ST (*t* = −2.189, *p* = 0.198, *d* = 0.33, 95% *CI* [−1.870, 0.209]) conditions, irrespective of age group. In the second case, perceived instability significantly increased during walking under DT compared to ST conditions (irrespective of arm movement strategy) in children (*t* = −3.267, *p* = 0.011, *d* = 0.50, 95% *CI* [−2.298, −0.202]) but not in young adults (*t* = 1.029, *p* = 1.000, *d* = 0.15, 95% *CI* [−0.609, 1.342]).

#### 3.1.4. Conscious Balance Processing

There were significant main effects for group, task, and arm, with a significant interaction between these factors (Figure 2D). Post hoc tests revealed significantly greater conscious processing of balance in young adults compared to children during walking under ST conditions with free (*t* = −5.148, *p* < 0.001, *d* = 1.31, 95% *CI* [−7.931, −1.751]) and restricted (*t* = −4.387, *p* = 0.002, *d* = 1.16, 95% *CI* [−7.544, −1.082]) arm movements. Further, conscious balance processing during walking under DT conditions with restricted arm movements was also significantly higher in young adults (*t* = −4.121, *p* = 0.004, *d* = 1.09, 95% *CI* [−7.245, −0.816]) than in children.

### 3.2. Walking Outcomes

#### 3.2.1. Gait Speed

There were significant main effects of task and arm, with participants walking slower during the DT (irrespective of arm movement strategy) and the restricted arm movement (irrespective of task) conditions. Further, we detected a significant task × arm interaction and group × task interaction (Figure 3A). In the first case, post hoc tests yielded a significant decrease in gait speed during walking with restricted compared to free arm movements under ST (*t* = 5.942, *p* < 0.001, *d* = 0.60, 95% *CI* [0.057, 0.155]) and DT (*t* = 3.917, *p* = 0.002, *d* = 0.30, 95% *CI* [0.016, 0.090]) conditions, irrespective of age group. In the second case, gait speed significantly decreased during walking under DT compared to ST conditions (irrespective of arm movement strategy) in children (*t* = 11.953, *p* < 0.001, *d* = 1.89, 95% *CI* [0.257, 0.410]) and in young adults (*t* = 8.572, *p* < 0.001, *d* = 1.26, 95% *CI* [0.151, 0.294]).

#### 3.2.2. Cadence

There were significant main effects for group and task, with a significant interaction between these factors (Figure 3B). Post hoc tests revealed a significant decrease in cadence during walking under DT compared to ST conditions (irrespective of arm movement strategy) in children (*t* = 7.874, *p* < 0.001, *d* = 1.51, 95% *CI* [0.439, 0.906]) and in young adults (*t* = 5.205, *p* < 0.001, *d* = 0.93, 95% *CI* [0.196, 0.632]).

### 3.3. Cognitive Task Performance

Irrespective of arm movement strategy, there was a main effect of group (*F* = 17.361, *p* < 0.001, $\eta_{p}^{2}$ = 0.24), with young adults (free: 0.46 ± 0.17, restricted: 0.44 ± 0.20) showing more calculations per second than children (free: 0.28 ± 0.16, restricted: 0.28 ± 0.13).

## 4. Discussion

The purpose of the present study was to examine the influence of different arm movement strategies on subjective task-related perception and walking outcomes while walking under ST and DT conditions in healthy children compared to young adults. First, and with respect to task conditions, we hypothesised that walking while simultaneously performing a cognitive task would impair both subjective and objective performance indices. The observed results supported our prediction. Precisely, balance confidence and conscious balance processing decreased, fear of falling increased and gait speed as well as cadence decreased during DT conditions. These findings are consistent with previous research [29,30] reporting deteriorations in both subjective task-related perception and balance outcomes during DT walking. For instance, Norouzian and colleagues [29] reported a significant increase in perceived anxiety when participants walked on flat ground while simultaneously performing a second task (i.e., auditory digit monitoring). Further, Beurskens and co-workers [30] showed that participants significantly decreased their gait speed and cadence when walking was combined with an arithmetic subtraction task. The observed deteriorations in subjective task-related perception and walking outcomes during DT walking can most likely be explained by limited cognitive capacities [3] and/or cognitive interference when two tasks share the same processing resources [4]. In other words, difficulties in allocating attentional resources to the two concurrently performed tasks or the inability to manage additional cognitive requests caused by limited information processing capacity may have provoked the detected DT-related deteriorations in subjective task-related perception and walking outcomes.

Second, and with regard to arm movement strategy, we hypothesised that DT-related deteriorations in subjective and objective performance indices would be lower when free arm movements are allowed. This assumption was confirmed by the detected task-by-arm interactions. More specifically, during DT walking with free versus restricted arm movements, the increase in perceived instability was less pronounced and gait speed was higher, while cognitive task performance remained unchanged. The latter finding is in line with earlier work [7,8] showing that deteriorations in DT walking performance were less severe when arms were freely moved. For example, da Silva Costa and colleagues [8] reported that the decrease in step speed while walking and simultaneously performing a sequential subtraction task was less pronounced under free compared to restricted arm movement conditions. These findings suggest that free arm movements are associated with improved performance under DT conditions across both objective and subjective indicators. In this regard, a previous study [31] suggested that the use of an ‘upper-body strategy’ for postural control is associated with several positive effects. From a biomechanical perspective, the authors argue that free arm movements produce stabilising impacts due to (a) enlargement of the moment of inertia [32], (b) generation of stabilising torques [33], (c) displacement of the centre of mass through changes in mass distribution [34], and (d) use of external support points [35]. Additionally, and from a cognitive perspective, free arm movements allow for (a) the use of more well-practiced motor patterns, (b) an increased awareness of the position and movement of one’s own limbs, and (c) the generation of proprioceptive feedback [31]. Accordingly, it seems plausible to argue that improved postural control—achieved using of free arm movements—may be associated with reduced cognitive demands during the task. These changes may have contributed to a greater sense of stability (i.e., a smaller increase in perceived instability) and a higher walking speed under DT conditions.

Third, and with respect to age group, we hypothesised that the compensatory effect of free arm movements on DT-related performance deteriorations would be less pronounced in children compared to young adults, since the former are still subject to ongoing development of postural control and cognitive processing capacities. In other words, despite free arm movements, the values between walking under ST and DT conditions should differ markedly in children but show hardly any differences in young adults. Our statistical analyses indicated a significant group × task × arm interaction for the subjective task-related perception but not for the walking outcomes. Contrary to our expectation, noticeable differences in conscious balance processing between walking under ST and DT conditions with free arm movements were observed in young adults (2.6 points), whereas hardly any changes were found in children (0.1 points). This implies that children’s conscious balance processing remained relatively stable across conditions, whereas young adults reported higher levels, particularly when arm movements were restricted. In addition, young adults showed more conscious balance processing than children under almost all conditions (except under the DT condition with free arm movements). The finding that children, compared to young adults, displayed lower and hardly altered conscious balance processing can be interpreted in two different ways. On the one hand, the children’s behaviour reflects an adequate cognitive response, as high values for conscious balance processing—that is, consciously thinking about movements that are normally “automatic” (such as gait)—impair attentional processing efficiency [28]. For example, this can lead to a “cautious gait,” characterised by slower, less efficient and more variable motor outcomes [36]. On the other hand, the response observed in children could represent an inadequate cognitive reaction because they may have been less “aware” of a potential DT-related loss of balance. Furthermore, consciously thinking about normally automatic gait control requires attentional and cognitive resources, while potential differences between children and young adults in the use of these resources cannot be conclusively determined.

The observation of more conscious balance processing in young adults during DT walking with free arm movements was not accompanied with poorer dynamic balance outcomes, as is typically reported [37]. This finding could be interpreted as pointing towards the assumption that young adults, compared to children, may possess sufficient attentional and cognitive resources to vary between automated and conscious postural control. Such a shift could potentially facilitate a more effective response to situations that challenge postural control or increase fall risk, such as walking under DT conditions [38], height-induced postural threat [28], or task constraints [39]. For children, however, it could be important to implement DT requirements in balance training in order to elicit adult-like adaptations (i.e., switch from automated to conscious postural control). However, this interpretation remains hypothetical and requires further investigation in future research.

## 5. Limitations

A limitation of the present study is that only one recorded trial per condition was collected, which might have affected reliability. In addition, repeated trials following task failure in a small number of participants may have introduced minor unequal practice effects. Consequently, future studies should include multiple trials per condition to assess intra-individual variability. Further, cadence was assessed visually without formal reliability evaluation. Lastly, several key outcome measures were based on subjective ratings, which may have been influenced by individual perception and reporting tendencies.

## 6. Conclusions

In conclusion, the present findings demonstrate that walking under DT conditions negatively affects both subjective task-related perception and walking outcomes in children and young adults, likely due to limited attentional resources and cognitive interference. Importantly, allowing free arm movements mitigated these deteriorations, indicating a compensatory effect. Age differences emerged in subjective task-related perception, i.e., children maintained relatively stable levels of conscious balance processing, whereas young adults showed higher levels and marked changes between walking under ST and DT conditions with free arm movements. This pattern could be interpreted as a possible hypothesis that young adults, compared to children, may vary between more automated and more conscious forms of postural control, whereas children may rely more strongly on automated processes. Overall, free arm movements appear to represent a simple and effective complementary ‘upper-body strategy’ to support postural control under cognitively demanding walking conditions.

## Figures and Tables

**Table 1:** Descriptive statistics showing group mean ± standard deviation values for all subjective task-related perception and walking outcomes for children and young adults by task conditions (single task vs. dual task) and arm movement strategies (free vs. restricted).

Parameter	Arm Strategy	Children (*n* = 26)		Young Adults (*n* = 30)	
Subjective task-related perceptions		ST	DT	ST	DT
Balance confidence (0–10)	Free	8.5 ± 1.7	6.9 ± 1.8	8.5 ± 1.6	7.0 ± 1.9
	Restricted	7.7 ± 1.7	5.6 ± 2.6	6.8 ± 1.7	5.4 ± 2.5
Fear of falling (0–10)	Free	0.8 ± 1.1	1.5 ± 1.7	1.2 ± 1.5	1.1 ± 1.5
	Restricted	1.8 ± 2.5	3.7 ± 2.5	2.7 ± 2.5	3.0 ± 2.6
Perceived instability (0–10)	Free	2.2 ± 2.0	2.8 ± 2.3	3.3 ± 2.9	2.6 ± 2.7
	Restricted	2.7 ± 2.1	4.5 ± 2.5	4.5 ± 2.8	4.5 ± 2.6
Conscious processing (4–24)	Free	8.7 ± 3.3	8.8 ± 4.2	13.5 ± 3.7	10.9 ± 3.8
	Restricted	9.7 ± 4.0	8.3 ± 3.8	14.0 ± 3.4	12.3 ± 3.5
Walking outcomes					
Gait speed (m/s)	Free	0.69 ± 0.24	0.31 ± 0.20	0.58 ± 0.17	0.34 ± 0.14
	Restricted	0.57 ± 0.20	0.28 ± 0.18	0.48 ± 0.15	0.27 ± 0.12
Cadence (*n*/s)	Free	1.67 ± 0.47	0.84 ± 0.43	1.28 ± 0.27	0.88 ± 0.26
	Restricted	1.47 ± 0.38	0.96 ± 0.91	1.16 ± 0.28	0.73 ± 0.26
Cognitive task performance					
Cognitive task performance	Free	N/A	0.28 ± 0.16	N/A	0.46 ± 0.17
	Restricted	N/A	0.28 ± 0.13	N/A	0.43 ± 0.20

DT: dual task; N/A: not available; ST: single task.

**Table 2:** Inferential statistics showing main and interaction effects of the repeated measures ANOVA for subjective task-related perception and walking outcomes.

Parameter	Group (Children vs. Young Adults)	Task (Single Task vs. Dual Task)	Arm (Free vs. Restricted)	Task × Arm	Group × Task	Group × Arm	Group × Task × Arm
Subjective task-related perception outcomes							
Balance confidence (0–10)	*F* = 0.360 *p* (*η*_p_^2^) = 0.551 (0.01)	***F* = 63.564** ***p* (*η*_p_^2^) < 0.001 (0.54)**	***F* = 50.674** ***p* (*η*_p_^2^) < 0.001 (0.48)**	*F* = 0.309 *p* (*η*_p_^2^) = 0.581 (0.01)	*F* = 0.918 *p* (*η*_p_^2^) = 0.342 (0.02)	*F* = 2.446 *p* (*η*_p_^2^) = 0.124 (0.04)	*F* = 1.111 *p* (*η*_p_^2^) = 0.297 (0.02)
Fear of falling (0–10)	*F* = 0.021 *p* (*η*_p_^2^) = 0.884 (0.00)	***F*** ** = 9.037** ***p*** ** (*η*_p_^2^) = 0.004 (0.14)**	***F*** ** = 48.456** ***p*** ** (*η*_p_^2^) < 0.001 (0.47)**	*F* = 3.480 *p* (*η*_p_^2^) = 0.068 (0.06)	***F*** ** = 6.974** ***p*** ** (*η*_p_^2^) = 0.011 (0.11)**	*F* = 0.064 *p* (*η*_p_^2^) = 0.801 (0.00)	*F* = 1.155 *p* (*η*_p_^2^) = 0.287 (0.02)
Perceived instability (0–10)	*F =* 1.743 *p* (*η*_p_^2^) = 0.192 (0.03)	*F* = 2.855 *p* (*η*_p_^2^) = 0.097 (0.05)	***F*** ** = 21.272** ***p*** ** (*η*_p_^2^) < 0.001 (0.28)**	***F*** ** = 4.653** ***p*** ** (*η*_p_^2^) = 0.035 (0.08)**	***F*** ** = 9.562** ***p*** ** (*η*_p_^2^) = 0.003 (0.15)**	*F* = 0.588 *p* (*η*_p_^2^) = 0.447 (0.01)	*F* = 0.451 *p* (*η*_p_^2^) = 0.505 (0.01)
Conscious processing (4–24)	***F*** ** = 20.949** ***p*** ** (*η*_p_^2^) < 0.001 (0.28)**	***F*** ** = 11.847** ***p*** ** (*η*_p_^2^) = 0.001 (0.18)**	***F*** ** = 5.135** ***p*** ** (*η*_p_^2^) = 0.027 (0.09)**	*F* = 0.245 *p* (*η*_p_^2^) = 0.623 (0.01)	*F* = 3.408 *p* (*η*_p_^2^) = 0.070 (0.06)	*F* = 1.870 *p* (*η*_p_^2^) = 0.177 (0.03)	***F*** ** = 6.964** ***p*** ** (*η*_p_^2^) = 0.011 (0.11)**
Walking outcomes							
Gait speed (m/s)	*F* = 1.499 *p* (*η*_p_^2^) = 0.226 (0.03)	***F*** ** = 212.839** ***p*** ** (*η*_p_^2^) < 0.001 (0.80)**	***F*** ** = 43.241** ***p*** ** (*η*_p_^2^) = 0.001 (0.45)**	***F*** ** = 6.709** ***p*** ** (*η*_p_^2^) = 0.012 (0.11)**	***F*** ** = 8.455** ***p*** ** (*η*_p_^2^) = 0.005 (0.14)**	*F* = 0.227 *p* (*η*_p_^2^) = 0.636 (0.00)	*F* = 2.240 *p* (*η*_p_^2^) = 0.140 (0.04)
Cadence (*n*/s)	***F*** ** = 7.666** ***p*** ** (*η*_p_^2^) = 0.008 (0.12)**	***F*** ** = 86.671** ***p*** ** (*η*_p_^2^) < 0.001 (0.62)**	*F* = 3.204 *p* (*η*_p_^2^) = 0.079 (0.06)	*F* = 2.764 *p* (*η*_p_^2^) = 0.102 (0.05)	***F*** ** = 4.914** ***p*** ** (*η*_p_^2^) = 0.031 (0.08)**	*F* = 0.857 *p* (*η*_p_^2^) = 0.359 (0.02)	*F* = 3.875 *p* (*η*_p_^2^) = 0.054 (0.07)

0.02 ≤ *η*_p_^2^ ≤ 0.12 indicates small, 0.13 ≤ *η*_p_^2^ ≤ 0.25 indicates medium, and *η*_p_^2^ ≥ 0.26 indicates large effects. Bold values indicate statistically significant differences (*p* ≤ 0.05).

## Data Availability

The original contributions presented in the study are included in the article; further inquiries can be directed to the corresponding author.

## Abbreviations

The following abbreviations are used in this manuscript:

ANOVA	Analysis of variance
DT	Dual task
N/A	Not available
ST	Single task
VAS	Visual analogue scale
